# Spectrophotometric Analysis and Determination of Anti-inflammatory Effect of Punica granatum and Woodfordia fruticosa in Subjects With Chronic Periodontitis – A Randomized Controlled Clinical Trial

**DOI:** 10.7759/cureus.50654

**Published:** 2023-12-17

**Authors:** Sujanamulk Bhavana, Sai Madhavi Nallamilli, Maneshwar Thippani, Namratha Gundapaneni, Yamini Sahitya, Vummadi Keerthi Reddy, Subhra Dey, Bharani Krishna Takkella, MP Venkata Prabhat, Nayanala Venkata Anusha

**Affiliations:** 1 Oral Medicine and Radiology, Drs Sudha and Nageswara Rao Siddhartha Institute of Dental Sciences, Vijayawada, IND; 2 Oral Medicine and Radiology, Mahaveer Institute Of Medical Sciences, Vikarabad, IND; 3 Chemistry, Anurag University, Hyderabad, IND; 4 Pediatric Dentistry, Yamini Dental Clinic and Implant Centre, Berhampur, IND; 5 Oral Medicine and Radiology, Kamineni Institute of Dental Sciences, Narketpally, IND; 6 Conservative Dentistry and Endodontics, All India Institute of Medical Sciences, Deoghar, IND

**Keywords:** spectrophotometry, woodfordia, punicagranatum, periodontal diseases, antiinflammatory agents, salivary alpha amylases

## Abstract

Introduction: Long-term use of chemical plaque control methods has led to adverse effects. In the current scenario, herbal mouthwashes have adequately remediated periodontal disease. Moreover, in the salivary interactome, biomarkers such as salivary amylase, a key protein secreted in the saliva, have been immensely useful in detecting the progression of periodontal disease. Therefore, we aimed to determine the anti-inflammatory effect of ethanolic crude extracts of *Woodordiafruticosa* leaf and *Punicagranatum* peel and to estimate salivary amylase levels in subjects diagnosed with chronic periodontitis.

Methodology: Ethanolic extracts of both plants were prepared using the soxhlet extraction method, and the obtained metabolites were confirmed by thin-layer chromatography. After a mouthwash was prepared, 100 subjects were randomly divided into two groups. Group I received *Punica* peel (Pgp) mouthwash, and Group II received *Woodfordia*leaf (Wfl). Clinical parameters such as probing depth and clinical attachment loss were recorded to determine the grades of periodontitis. Unstimulated whole saliva was collected, and amylase levels were analyzed at three-time intervals using spectrophotometric assay.

Results: For both groups, the mean reduction in salivary amylase levels was calculated at baseline after 1 hour and 1 week of using the mouthwash. A statistically highly significant reduction was seen with (p=0.000*) at the 1-hr interval in Group I.

Conclusion: The anti-inflammatory effect was more effective with Pgp mouthwash when compared to Wfl mouthwash.

## Introduction

Periodontal diseases are chronic inflammatory disorders, the most common of which are gingivitis and periodontitis. They result from different aerobic and anaerobic bacteria and their products, leading to the destruction of tooth-supporting tissues [1]. Gingivitis is deﬁned as a plaque-induced inﬂammation of the marginal gingiva, whereas periodontitis is a chronic inﬂammation that causes the destruction of the connective tissue and surrounding alveolar bone of the tooth [2]. Several etiological factors, such as systemic diseases, habits, social factors, and psychological stress, have been implicated as risk factors for the onset and progression of periodontal diseases [3]. Today, the ﬁeld of salivanomics, which has advanced significantly in recent decades, has focused on the biomarker research and analysis of nucleic acids. The saliva of humans is a rich reservoir of proteins and peptides; it gathers more than 3652 proteins and 12,562 peptides and shares almost 51% of the proteins and 79% of the peptides contained in the plasma [4,5]. Owing to the discovery of many biomarkers in saliva, there has been scope for innumerable therapeutic targets to solve the problems of oral diseases and associated systemic manifestations. Biomarker research in periodontology has recently been developed as a high-impact diagnostic, significantly affecting clinical decision-making, patient outcomes, and healthcare economics. There is ample evidence that has proven their role in early disease detection and progression of oral diseases [6]. The salivary biomarkers have proven beneficial in monitoring health status, disease susceptibility, and progression of periodontal diseases. Some nonspeciﬁc proteins are altered in patients with periodontal disease, including albumins, amylases, mucins, and lactoferrins [7]. However, the salivary enzyme alpha-amylase is said to be the key protein, accounting for about 60% of all proteins produced by salivary glands. Alpha amylase is chiefly involved in modulating bacterial adhesion, thus inhibiting the growth of microorganisms on intraoral surfaces [8]. Various studies show reduced salivary antimicrobial and defense properties among periodontally compromised patients. Various authors, including Boras et al. (2012) [9], Kejiriwal et al. (2014) [10], Acquier et al. (2015) [11], Carolina Dyah et al. (2017)[12], Neha et al. (2018) [8], and Lorenzo Posso et al.(2018) [7] have demonstrated the relationship between salivary amylase levels and chronic periodontitis.

Moreover, chemotherapeutic agents such as essential oils, chlorohexidine, and triclosan have led to certain side effects such as staining teeth and tongue, taste disturbance, and adverse effects on the oral mucosa. Hence, an alternative must be identified, and the focus of periodontal disease research must be shifted to botanical sources such as herbal products [13]. The herbal mouthwashes contain various active agents with phytoconstituents such as flavonoids, glycosides, catechins, tannins, terpenoids, and sterols that have proven effective through their antimicrobial, anti-inflammatory, and antioxidant properties. Although numerous herbal mouthwashes have been tested, the clinical effects and the results of existing literature have been inconsistent [14]. Moreover, the anti-inflammatory effects of Lythraceae have not received much attention, particularly on some of its species, like Punicagranatum (Pg) and Woodfordfruticosa (Wf).

Woodfordfruticosa, also called fire flame bush, is an evergreen shrub grown in tropical and subtropical regions of India. Various biological activities, such as antimicrobial, antioxidant, immunomodulatory, analgesic, and anti-inflammatory, have been reported. An array of phytoconstituents like flavonoids, terpenoids, alkaloids, and saponins in the leaves of Wf are known to exhibit anti-inflammatory activity [15].

Punicagranatum also called the pomegranate, has been used in traditional medicine to treat various ailments. In particular, Punica peel is known to have a range of therapeutic, anti-inflammatory, anti-mutagenic, and anti-fungal properties [16]. Phytocompounds such as tannins, alkaloids, flavonoids, and phenolic acids are thought to be the major contributors to the therapeutic properties of Pg peel [17].

In particular, when we tried to search the literature for the anti-inflammatory potential of Woodfordia using popular research engines such as Embase, Medline, Science Citation Index, NIH public access, Pubmed, and the Cochrane Database of Systematic Reviews, we found no existing literature comparing the anti-inflammatory effects of Punica and Woodfordia by assessing the salivary amylase levels in chronic periodontitis; therefore, our research is the first of its kind to focus particularly on this clinical aspect.

## Materials and methods

The current study was conducted in the Department of Oral Medicine and Radiology, Dr. Sudha and Nageswararao Siddhartha Institute of Dental Sciences between 2021 and 2022. The study procedures adhered to CONSORT guidelines and were by guidelines of the Declaration of Helsinki, revised in 2013. The Institutional Ethical Committee approved the study protocol. Informed consent was taken from all the subjects willing to participate in the study. This study procedure was a continuation of our previous study published in the Journal of Clinical and Diagnostic Research in 2016, with IEC: REF/2016/03/11053 and the current IEC ref no REF/2022/02/1099 given for the Anti-inflammatory activity which was a continuation of study.

Ideally, 51 patients were there in Group I, and in Group II, with 56 subjects after taking the first sample, seven dropouts were there, with 50 in each group. Hence, the final sample was 100, consisting of 50 in each group aged 35-60, including male and female participants. They were randomly divided into two groups based on a coin toss. Based on periodontal probing, patients with mild to moderate periodontitis, those with pocket probing depth of ≥ 5-7mm and clinical attachment loss of ≥ 3mm, and subjects without periodontal treatment in the past six months were included in the study. Group I (50 patients) received the Pgp mouthwash, and Group II (50) received the Wfl mouthwash in a double-blinded, randomized, controlled clinical trial. Both the participants and the physician were blinded to the allocated product.

Exclusion criteria included those who had smoking habits or had systemic diseases, autoimmune diseases, or anti-inflammatory drugs; those who had undergone periodontal therapy or used mouthwash for less than six months; and pregnant or breastfeeding individuals.

Extraction, isolation, and purification of bioactive compounds: The dried leaves of Wfl and peel of Pgp (30gm each) were each put in a mechanical grinder, and the grounded powder was subjected to extraction using 200ml of 70% ethanol by soxhlet extractor at a temperature of 60 °C for 4 hrs. The obtained extracts were filtered using Whatman filter paper No. 3 and concentrated under reduced pressure in a rotary evaporator to remove the solvent [14]. 

Phytochemical analysis

Phytochemical screening was done, and biochemical tests were conducted. Quantitative phytochemical analysis was done to check the presence of alkaloids, flavonoids, saponins, steroids, glycosides, and carbohydrates in Woodfordia leaf and flavonoids, tannins, steroids, alkaloids, terpenoids in Punica granatum peel. Secondary metabolites, such as glycosides and carbohydrates, were found in Wfl, and flavonoids, steroids, and terpenoids were found in Pgp. To confirm the presence of these phytoconstituents, high-performance thin-layer chromatography was performed, where 10µl of alcoholic extract dissolved in ethanol was applied in the form of a band of width 6mm with a 100µl sample syringe to a pre-coated silica gel aluminum plate 60 F254 ( 5x10) with 250µm thickness using a CAMAG Linomat 5 sample applicator. The composition of the mobile phase was ethanol: isopropyl alcohol: triethyl amine (5:4:1) [14].

Rf = A/B

A= distance between the point of application and the central point of the spot being examined; B= distance between the point of application and the mobile phase point.

The plate was observed in daylight under UV light (254 and 366 nm). After each observation, the central point of spots was marked with a needle. The retention factor (Rf) was calculated by following the formula of Kumar S et al. (2013) [18]. Later, the identified metabolites were separated using thin-layer chromatography.

Preparation of mouthwash

After the above procedure, 0.8 gms of Pgp and Wfl were superadded with glycerol 10ml, L-menthol 0.2gm, peppermint oil 0.05gm and citric acid 0.2gms. Subsequently, sufficient water was added to make a total volume of 100 ml of each prepared mouthwash [14].

Meanwhile, the subjective satisfaction of taste and smell upon mouthwash was assessed using a 9-point hedonic scale [19]. Positive numbers were interpreted as moderate to strong satisfaction, and negative numbers as weak satisfaction. The burning sensation was assessed using the Visual Analog Scale, and allergic reactions to the prescribed mouthwash were also recorded at all time intervals.

Collection of saliva

Subjects were comfortably seated in an upright position on a dental chair. Unstimulated whole saliva was collected between 11 a.m. and noon to avoid diurnal variations. Participants were instructed not to eat, drink, or smoke one hour before the sample collection. The salivary amylase levels were estimated at three intervals. At baseline, the subjects were asked to swish using physiological saline; later, they were randomly allocated to swish either of the mouthwashes along with their routine oral hygiene instructions. The samples were collected at 1-hr and 1-week time intervals. It is usually recommended to wait at least 60 min to allow the active ingredients of mouthwash to work effectively. Hence, we collected the sample after 1 hr.

Moreover, to see the long-term efficacy of tested mouthwashes, we took samples after 1 week; the washout period was not considered in our study as the subjects were asked to use the same mouthwash throughout the week along with oral hygiene instructions. The subjects were instructed to flex their head and neck forward to allow the passive flow of saliva to accumulate on the floor of the mouth and were made to spit in sterile containers. The sample collection was 6 minutes for each subject. After incubation, samples were centrifuged for 20 min at 3000 rpm, with a dilution factor 1:10. The resulting supernatant was then used to estimate amylase.

Estimation of salivary amylase

To determine the salivary amylase activity, the Liquimax-α amylase SLR kit (σ Six Sigma, Ltd.) was used. Salivary amylase activity was detected by the 2-chloro-4-nitrophenyl-α-D-maltotrioside (CNPG3) method. The kit contains 96 well plates, a spectrophotometric plate reader, a phosphate buffer diluent for saliva samples, and a chromogenic substrate (2-chloro-p-nitrophenol linked with maltotriose) acting on α-amylase. To perform the test, 20µl of saliva sample was taken, mixed with 1000µl of the reagent, and incubated at 37oC. The salivary supernatant was put into the same test tube, and the absorbance was measured. The presence of amylase resulted in a yellow color; the sample was then analyzed spectrophotometrically at 405nm [12].

Statistical analysis

The analysis was performed using SPSS version 22. The quantitative variables were expressed as mean and standard deviation. For comparison of the two means, a student unpaired t-test was performed. For all tests, the probability value was assessed as p ≤ 0.05, which was considered a significant association at a 5% significance level, where p ≤ 0.01 was considered highly significant and p ≥ 0.05 was insignificant.

## Results

The mean values of salivary amylase levels were calculated at different time intervals in Group I and Group II. At baseline, there was no difference, although after 1 hour of using Pgp mouthwash, a drastic reduction of amylase levels was seen, and the p-value was highly significant (p = 0.001)*; however, after one week of using both mouthwashes, there was no difference noted with regards to reduction of salivary amylase levels. The details are shown in Table 1.

**Table 1:** Comparison of mean values of salivary amylase levels in group I and group II at different time intervals SD- Standard deviation, N- total number, P- Probability value

Time intervals	Groups	N	Mean	SD	p-value
Baseline	Group I	50	172.89	66.169	0.647
	Group II	50	178.75	65.644	
After 1 hour	Group I	50	6.091	5.4356	0.000^*^
	Group II	50	131.161	62.1063	
After 1 week	Group I	50	125.05	68.016	0.821
	Group II	50	122.43	48.670	

**Figure 1:**
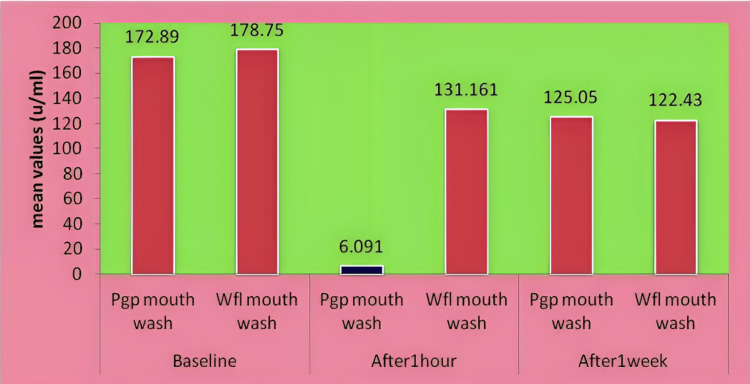
Graph representing mean values of salivary amylase levels in both the groups at different time intervals Pgp- Punica granatum peel, Wfl- Woodfordia fruticosa leaf

In Figure 1, the graph represents the mean values of salivary amylase levels in both groups at different time intervals. At baseline, no difference was seen, although a drastic reduction of amylase levels with a mean value of 6.091 was seen after using the Pgp mouthwash. No difference was seen in either group after one week of using the mouthwash, as summarized in the graph.

Spectral analysis of Punicagranatum

**Figure 2:**
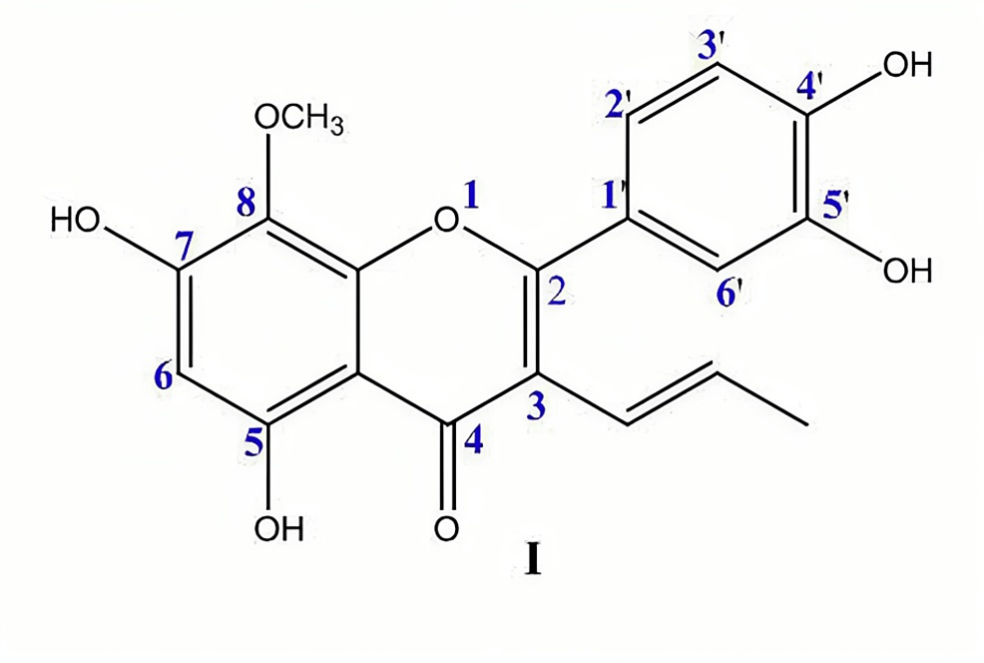
Flavonoid structure of Punica granatum peel by spectral analysis OCH3 – Methoxy group; OH – Hydroxide; HO – Hydroxyl group; O – Oxygen

The interpretation of spectral data was based on physicochemical properties and comparison with literature data. The functional groups, number of protons and carbons in the structure, and molecular weight of the compound were recorded by IR (infrared spectra), H1NMR (proton nuclear magnetic resonance spectroscopy), C13NMR (carbon-13 nuclear magnetic resonance), and mass spectrometry, respectively. The spectral data revealed a flavone skeleton and the available data for Pgp suggested the presence of flavonoids. The details are described in Table 2 and represented in Figure 2.

**Table 2:** Quantitative analysis of Punicagranatum peel showing chemical characterization C – Carbon atom; δ – Delta scale showing chemical shifts; 13C – Carbon-13; ppm – Parts per million; 1H – Hydrogen-1; CH – methane; CH-OH – Hydroxy methyl group; OCH3 – Oxygen methyl group (Methoxy group).

Position	C atom	δ ^13^C (ppm)	δ ^1^H (ppm)
2	C	124.76	-
3	C	148.92	-
4	C=O	172.25	-
4a	C	134.23	-
5	C-OH	151.82	11.58
6	CH	125.73	7.9
7	C-OH	161.82	11.85
8	CH		-
1^1^	C	134.51	-
2^1^	CH	129.42	7.3
31	CH-OH	148.01	8.10-
41	CH-OH	148.9	9.32
5^1^	CH	126.09	7.15
6^1^	CH	125.79	7
1”	CH=	120	2.56
2”	=CH	113.3	2.5
3”	CH_3_	24.4	1.55-146
9	OCH_3_	55.43	3.36-3.50

## Discussion

There are 564 common proteins in five body fluids (saliva, plasma, CSF, urine, and amniotic fluid) [20]. (Among the salivary interactome of all the proteins, a nonspecific biomarker, namely salivary amylase, has proved to be altered in patients with chronic periodontitis compared with healthy patients [21]. Salivary alpha-amylase is a calcium-containing metalloenzyme known as 1,4-α-D-glucanglucanohydrolase that occurs predominantly in salivary fluid and pancreatic juice [22]. Our study results demonstrated that salivary amylase levels were significantly higher in subjects with chronic periodontitis. These results were by other studies conducted by Sanchez GA (2013) [23], Ahmadi Motamayel F et al. (2017) [24], and Neha T et al. (2018) [8], where a positive correlation was observed with regards to increased amylase levels in subjects with chronic periodontitis.

Kejiriwal et al. (2014) [10] estimated the levels of salivary mucin, amylase, and total protein in patients with gingivitis and chronic periodontitis where all the biomarkers were gradually increased, indicating as an important biochemical parameter in the inflammation of periodontium. Similarly, Neha et al. [8] confirmed that salivary amylase was increased in subjects with chronic gingivitis and chronic generalized periodontitis. The other supported studies with the same conclusion were addressed in those conducted by Hady (2012) [25] and Hernandez-Castameda AA et al. (2015) [26].

The rationale behind these findings is that the secretion of proteins such as alpha-amylase is controlled directly under stimulation of an inflammatory process in addition to the infectious process and stress-loading conditions where the sympathetic nervous system is activated. This sequence, therefore, increases saliva's potential against the accumulation of plaque-derived substances and inflammatory products involved in periodontitis [24]. The other reason is that the production of pro-inflammatory and anti-inflammatory mediators might be modified by catecholamines, which influence the disease activity [3]. It also has been proved that salivary amylase binds to microorganisms such as A. actinomycetans or P. gingivalis, which interferes with bacterial adherence and biofilm formation, demonstrating that these high levels can serve as important defense molecules essential for innate immunity. This has been supported by Hariharan et al. [3], who states that a special process of local immune AA Actinobacillus actinomycetemcomitans is responsible for defense against chronic periodontal disease. Comparative studies of amylase levels about the views of different authors were made where Sanchez et al. [23] stated the origin of salivary proteins had been derived from salivary glands and GCF, although Ahmadi et al. [24] affirmed that there may be a different origin of amylase produced by salivary glands.

To the best of our knowledge, the anti-inflammatory effect of Punica and Woodfordia by estimating salivary amylase levels has not been documented; hence, in the current study, we compared the anti-inflammatory effect of Punica and Woodfordia with an estimation of salivary amylase in subjects with chronic periodontitis. In the current study, there was a tremendous decrease in amylase levels after 1 hour of using Pgp mouthwash over 1 week time interval. Our results are from previous studies done by Dilsilvestro et al. [27], where a significant reduction in salivary proteins was seen with Pgp extracts. Bhadbade et al. [28] conducted a clinical trial to determine the amount of plaque accumulation for five days using Punica granatum, chlorohexidine, and placebo and found that Pg had significantly less accumulation and prevented plaque as effectively as chlorohexidine. Their research findings are in accordance with the current clinical trial where there was a remarkable decrease in amylase levels with Pg. The mechanism of action behind the anti-inflammatory effect is explained by several authors where Lee et al. [29] determined that among the four hydrolysable tannins in their study, granatin in Punica displayed the ability to inhibit nitric oxide and also had the strongest PGE2 inhibitory effect in vitro and in vivo; supporting that granitin could be used as a standard marker for the anti-inflammatory effect of Punica.

Similarly, Sastravaha et al. [30] observed a reduction of IL-1β and IL-6 using Pg extracts. Pg is also effective in reducing TNF-α by interfering with the activity of elastase, metalloproteinase-3, and myeloperoxidase. The anti-inflammatory mechanism of Pg may be explained by its ability to inhibit NF-κB activity by blocking the cell-signaling pathways.

Moreover, pomegranate seed oil also can inhibit cyclooxygenase 1, 2, and lipoxygenase enzymes, which play a key role in inflammation. In the current study, flavonoids in Pg showed a potent anti-inflammatory effect. It is thought that flavonoids block the synthesis of inflammatory mediators such as IL-1 TNF-α, NO, and COX -2 and suppress VEGF and ICAM-1 expression, along with activating STAT3, NFkB, NLRP3 inflammasome, and MAP kinases pathways. However, flavonoids have poor water solubility and inadequate permeability and bioavailability, thus requiring high doses.

The anti-inflammatory activity of Woodfordia fruticosa extracts has been studied in animals, although no human studies have been conducted to date. In the current study, there was a greater decrease in amylase levels at 1 week of using Wf mouthwash than at the 1-hour time interval. The anti-inflammatory effect of different Wf stems and analgin extracts on albino Wistar rats was conducted where anti-inflammatory efficacy was at par with analgin with aqueous Wf stem. Several studies have been conducted on the ayurvedic concept of Wf flowers and tested for anti-inflammatory activity compared with diclofenac, where a mild variation could be seen between the anti-inflammatory activity of Wf flowers and diclofenac. Hence, Wf can definitely be used as an alternative.

Suggestions and limitations

The current study has limitations, as there was no control group, and we only compared the efficacy of two herbal mouthwashes. The sample size was small due to problems with follow-ups and patient cooperation. Future studies are recommended, such as prospective clinical trials with control. Moreover, long-term follow-up would help assess the herbal mouthwash's efficacy and side effects.

## Conclusions

The anti-inflammatory effect was more effective with the Pgp mouthwash than with the Wfl mouthwash. There was a remarkable decrease in salivary amylase levels at the 1-hour interval with Pgp mouthwash use. Furthermore, the advances in the discovery of new anti-inflammatory targets may help develop new formulations by encouraging researchers to work on the isolation and characterization of phytoconstituents such that new drug leads help identify the candidate drugs for the treatment of periodontal diseases. 
